# Increasing Number of Scarlet Fever Cases, South Korea, 2011–2016

**DOI:** 10.3201/eid2401.171027

**Published:** 2018-01

**Authors:** Jong-Hun Kim, Hae-Kwan Cheong

**Affiliations:** Sungkyunkwan University School of Medicine, Suwon, South Korea

**Keywords:** scarlet fever, notification, incidence, bacteria, respiratory infections, streptococci, group A *Streptococcus*, outbreak, surveillance, Korea, Republic of Korea, South Korea

## Abstract

The increasing number of reported scarlet fever cases during 2011‒2016 in the National Notifiable Infectious Disease database in South Korea occurred because of increased overall reporting and expanded reporting criteria rather than because of increasing scarlet fever incidence. Further increases are anticipated because of other expansions in reporting requirements.

Studies suggest that scarlet fever incidence has been increasing in South Korea (Republic of Korea) and other countries of East Asia since 2011 (*1**–**3*). The report describing increased numbers of scarlet fever cases in South Korea was based on National Notifiable Infectious Disease (NNID) surveillance data, which comprises cases reported through an electronic system to the Korea Centers for Disease Control and Prevention (*4*). In South Korea, scarlet fever is categorized as a group 3 NNID, which requires continuous surveillance and the establishment of control measures against possible outbreaks because of the risk for intermittent epidemics. Medical professionals who work for medical institutions are required to report cases to the local public health office. Reported data are reviewed by the local health center staff and submitted to the health authority of the province and Korea Centers for Disease Control and Prevention through an electronic reporting system (*4*). However, despite the law, the reporting rate of infectious disease by medical institutions has been low. Assessing the sensitivity of this reporting system for detecting scarlet fever cases was the goal of this report.

South Korea has a single-payer public health insurance system with universal coverage; the National Health Insurance Service is the insurer, and the Health Insurance Review and Assessment Service (HIRA) reviews payments. Using HIRA data, institutions can obtain information on the diagnoses of diseases and treatments for the entire population (*5*).

According to HIRA data, 14,550 patients in 2011 and 15,533 patients in 2013 had scarlet fever diagnoses (International Statistical Classification of Disease and Related Health Problems, Tenth Revision code A38) (Technical Appendix Table 1). The number of scarlet fever cases decreased in the following 2 years and then increased again in 2016 to 13,261 cases (*6*). However, according to the NNID surveillance data, 406 cases of scarlet fever were reported in 2011, with the number of cases increasing with time, ending with 11,911 cases in 2016 (Figure), and most patients being young (<10 years of age; online Technical Appendix Table 2) (*7*). The inconsistencies between these 2 databases indicates that the number of in-hospital diagnoses of scarlet fever has been roughly constant, but the reporting rate of diagnosed scarlet fever increased markedly from 2.8% in 2011 to 89.8% in 2016.

**Figure:**
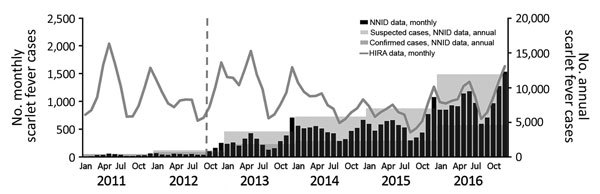
Incidence of scarlet fever determined using NNID and HIRA data, South Korea, 2011‒2016. The blue dashed line indicates the starting point for NNID reporting using the expanded criteria. HIRA, Health Insurance Review and Assessment Service; NNID, National Notifiable Infectious Disease.

Patients with NNIDs fall into the following 3 categories: confirmed case-patient, a person with compatible clinical symptoms who was positive for a group A *Streptococcus* by laboratory tests; suspected case-patient, a person with compatible clinical symptoms who was not tested or not positive for the pathogen by laboratory tests; and pathogen carrier. In South Korea, the reporting criteria for scarlet fever were limited to confirmed case-patients until September 2012, after which the criteria expanded to include suspected case-patients. This change contributed to the sharp increase of reported scarlet fever cases in the South Korea NNID database in 2013. This sharp increase was also facilitated by another factor: medical institutions and government agencies were aware of the poor NNID reporting rate and made efforts to improve them around this time. In a previous study, the incidence of scarlet fever in South Korea was reported to have increased rapidly on the basis of NNID data (*3*). However, considering the background of the reporting system, we believe that HIRA data, rather than the NNID database, better reflect the rate of infection in South Korea.

We report a discrepancy between the number of patients with scarlet fever diagnoses in hospitals and the numbers reported in the NNID system. We speculate that the rapid increase in the number of reported cases of scarlet fever in South Korea was not caused by an increase in the number of cases but was due to external factors, such as a drastically increased reporting rate and the inclusion of suspected cases due to the expanded reporting criteria. However, we do not completely exclude the possibility that the increased number of confirmed cases reflects an increased number of patients with scarlet fever because the number of confirmed cases, not just the overall cases, in the NNID database has been steadily increasing. Rapidly expanding private health insurance coverage, which provides additional funding, might be increasing access to healthcare for some patients and, thus, the number of persons seeking treatment for scarlet fever. In 2016, the Infectious Diseases Control and Prevention Act was amended to expand the reporting institutions for NNID from medical institutions to inspection agencies, concurrent with the government’s efforts to increase the reporting rate of NNID. Therefore, the reporting rate of NNID in South Korea is expected to be much higher in the future.

**Technical Appendix.** Comparison of cases reported to the National Notifiable Infectious Disease database and the Health Insurance Review and Assessment Service and age distribution of patients with scarlet fever.

## References

[1] Mahara G, Chhetri JK, Guo X. Increasing prevalence of scarlet fever in China. BMJ. 2016;353:i2689. 10.1136/bmj.i2689 27188472

[2] Lau EH, Nishiura H, Cowling BJ, Ip DK, Wu JT. Scarlet fever outbreak, Hong Kong, 2011. Emerg Infect Dis. 2012;18:1700–2. 10.3201/eid1810.120062 23017843 PMC3471616

[3] Park DW, Kim SH, Park JW, Kim MJ, Cho SJ, Park HJ, et al. Incidence and Characteristics of Scarlet Fever, South Korea, 2008-2015. Emerg Infect Dis. 2017;23:658–61. 10.3201/eid2304.160773 28322696 PMC5367408

[4] Park S, Cho E. National Infectious Diseases Surveillance data of South Korea. Epidemiol Health. 2014;36:e2014030. 10.4178/epih/e2014030 25420951 PMC4272235

[5] Cheol Seong S, Kim YY, Khang YH, Heon Park J, Kang HJ, Lee H, et al. Data Resource Profile: The National Health Information Database of the National Health Insurance Service in South Korea. Int J Epidemiol. 2017;46:799–800. 10.1093/ije/dyw253 27794523 PMC5837262

[6] Health Insurance Review and Assessment Service. Disease classification statistics [cited 2017 Aug 9]. http://opendata.hira.or.kr/op/opc/olap4thDsInfo.do#none

[7] Korea Centers for Disease Control and Prevention. Disease web statistics system [cited 2017 Aug 9]. https://is.cdc.go.kr/dstat/jsp/stat/stat0001.jsp

