# Supplementary material for: Increasing Number of Scarlet Fever Cases, South Korea, 2011–2016
Source: Emerg Infect Dis. 2018 Jan;24(1):172–3. doi: 10.3201/eid2401.171027 (PMC5749449; doi:10.3201/eid2401.171027)
Supplement: Supplementary file 1 — Technical Appendix. Comparison of cases reported to the National Notifiable Infectious Disease database and the Health Insurance Review and Assessment Service and age distribution of patients with scarlet fever. [file 17-1027-Techapp-s1.pdf]

Article DOI: https://doi.org/10.3201/eid2401.171027

Increasing Number of Scarlet Fever Cases, South Korea, 2011–2016

Technical Appendix

Technical Appendix Table 1. Comparison of confirmed and suspected cases reported through NNID database with cases reported through HIRA, South Korea, 2011–2016*

	NNID				
Year	Total	Confirmed, n (%)	Suspected, n (%)	HIRA	NNID/HIRA, %
2011	406	406 (100.0)	0 (0)	14,550	2.8
2012	968	713 (73.7)	255 (26.3)	12,723	7.6
2013	3,678	1,855 (50.4)	1,823 (49.6)	15,533	23.7
2014	5,809	2,312 (39.8)	3,497 (60.2)	11,656	49.8
2015	7,002	2,356 (33.6)	4,646 (66.4)	9,666	72.4
2016	11,911	4,598 (38.6)	7,313 (61.4)	13,261	89.8

*NNID, National Notifiable Infectious Disease; HIRA, Health Insurance Review and Assessment Service.

Technical Appendix Table 2. Age distribution of patients with reported cases of scarlet fever in the NNID database, South Korea, 2011–2016*

	No. (%)					
Age group, y	2011	2012	2013	2014	2015	2016
0–4	169 (41.6)	402 (41.5)	1,619 (44.0)	2,683 (46.2)	3,314 (47.3)	5,430 (45.6)
5–9	228 (56.2)	542 (56.0)	1,937 (52.7)	2,920 (50.3)	3,416 (48.8)	5,972 (50.1)
10–14	8 (2.0)	20 (2.1)	104 (2.8)	184 (3.2)	221 (3.2)	334 (2.8)
≥15	1 (0.2)	4 (0.4)	18 (0.5)	22 (0.4)	51 (0.7)	175 (1.5)

*NNID, National Notifiable Infectious Disease.
